# Flat epitaxial quasi-1D phosphorene chains

**DOI:** 10.1038/s41467-021-25262-7

**Published:** 2021-08-27

**Authors:** Wei Zhang, Hanna Enriquez, Yongfeng Tong, Andrew J. Mayne, Azzedine Bendounan, Alex Smogunov, Yannick J. Dappe, Abdelkader Kara, Gérald Dujardin, Hamid Oughaddou

**Affiliations:** 1 Université Paris-Saclay, CNRS, Institut des Sciences Moléculaires d’Orsay, Orsay, France; 2 TEMPO Beamline, Synchrotron SOLEIL, Gif-sur-Yvette, Cedex France; 3 Université Paris-Saclay, CNRS, CEA, Service de Physique de l’Etat Condensé, Gif-sur-Yvette, France; 4 Department of Physics, University of Central Florida, Orlando, FL USA; 5 Département de Physique, CY Cergy Paris Université, Cergy-Pontoise, Cedex France

**Keywords:** Surface chemistry, Synthesis and processing

## Abstract

The emergence of peculiar phenomena in 1D phosphorene chains (P chains) has been proposed in theoretical studies, notably the Stark and Seebeck effects, room temperature magnetism, and topological phase transitions. Attempts so far to fabricate P chains, using the top-down approach starting from a few layers of bulk black phosphorus, have failed to produce reliably precise control of P chains. We show that molecular beam epitaxy gives a controllable bottom-up approach to grow atomically thin, crystalline 1D flat P chains on a Ag(111) substrate. Scanning tunneling microscopy, angle-resolved photoemission spectroscopy, and density functional theory calculations reveal that the armchair-shaped chains are semiconducting with an intrinsic 1.80 ± 0.20 eV band gap. This could make these P chains an ideal material for opto-electronic devices.

## Abstract (web summary)

Similarly to graphene, attempts to fabricate phosphorene by epitaxy or starting from a few layers of bulk black phosphorus have failed so far. Here, the authors present a controllable bottom-up approach to grow atomically thin, crystalline 1D flat phosphorus chains on a Ag(111) substrate.

## Introduction

Two-dimensional (2D) materials have been at the forefront of research over the past decade. Following the discovery of graphene^1,2^, many different 2D materials have appeared since: silicene^3,4^, transition-metal dichalcogenides^5^, germanene^6^, borophene^7,8^, and phosphorene^9,10^. While the remarkable properties of these 2D materials are now known, reducing the dimensionality reveals physical properties that help to overcome certain limitations. However, the fabrication of chains based on 2D materials has proven to be a huge challenge. Many attempts to shape 2D materials into quasi-one-dimensional (quasi-1D) structures have been made, notably in the case of graphene, where lithography and etching techniques were first applied to make nanoribbons (NRs)^11^. Unfortunately, this approach is severely limited by the lack of control over the width, length, and disordered structure of the NRs that could be obtained^12^, preventing any reproducible measurements or implementation into larger-scale electronic circuits. This was overcome by the use of a bottom–up chemical synthesis approach that achieves perfect control of the graphene NR^13^. An energy gap is induced in the narrow graphene NRs that does not otherwise exist in 2D graphene, thus enabling a field-effect transistor to be operated^12,14^.

Among 2D materials, black phosphorus (BP) is composed of a 2D lattice of *sp*^3^ hybridized phosphorus atoms arranged in a buckled sheet. BP has several properties that distinguish it from other 2D materials; in particular, it has a strong in-plane anisotropy^15^ and an intrinsic direct band gap that can be tuned from 1.80 eV for a monolayer (ML)^16^ to 0.35 eV for bulk BP crystals^9^. It is suitable for nanoscale device applications because of a high on/off ratio (10^5^) and large carrier mobility (~1000 cm^2^/V/s)^9,17,18^.

There is a large family of phosphorus allotropes, which can be subdivided into different categories. Each category exhibits vastly different crystalline structures and properties. White phosphorus contains four phosphorus atoms arranged in P_4_ tetrahedrons^19^. Although white phosphorus is one of the most common phosphorus allotropes, it is highly unstable under ambient conditions^20^. However, red phosphorus is a relatively stable allotrope of phosphorus with a polymeric chain-like structure^21^. Black phosphorus is the most stable allotrope^22^. Since each phosphorus atom has five valence electrons, each atom is bonded to three adjacent phosphorus atoms with *sp*^3^ hybridized orbitals, resulting in a puckered honeycomb structure^23^, in which the single atomic layers are stacked together by weak van der Waals interactions instead of covalent bonds. BP possesses two distinctive lattice constants for the armchair (4.37 Å) and zigzag edges (3.31 Å), respectively. Blue phosphorus, another unique and stable phase of phosphorus, was only grown by the molecular beam epitaxy (MBE) using black phosphorus or InP as precursor^24,25^, presenting a buckled honeycomb structure. This structure presenting zigzag puckering can be obtained by a specific translational dislocation of  black phosphorene^26^. Recently, pure violet phosphorus has been reported^27^. It is composed of tubular strands with a monoclinic crystal structure^28^. Fibrous phosphorus also belongs to the family of phosphorus allotropes, composed of 1D tubular layers held together by van der Waals interactions^29,30^.

Theoretical studies^31–40^ have predicted that reducing the dimensionality of a single atomic layer of phosphorus into 1D P chains should reveal peculiar phenomena, in particular the Stark effect^34^, the Seebeck effect^35^, room temperature (RT) magnetism^36^, and topological phase transitions^37^.

Top–down methods have been reported for P NRs (PNRs) from bulk black phosphorus^41–43^. Using ionic scissoring of macroscopic black phosphorus crystals, high-quality individual PNRs have been produced^41^. Electro-chemical exfoliation following BP synthesis has been also reported to give phosphorene sheets and NRs^42^. However, both the lithography and electro-chemical techniques are not yet able to produce or control the width of the narrowest PNRs. The controlled fabrication of perfectly defined ribbons has been achieved for graphene by the polymerization of molecular precursors^13^. However, until now this method is still not yet transferable to phosphorene due to its stronger sensitivity to air^44^ and chemical reactivity^45,46^ compared to graphene^47^. In the literature, most studies have looked at 3–10 nm-wide quasi-1D NRs or stripes that can form 2D arrays.

Here we present the synthesis of self-assembled crystalline phosphorene-like chains on a Ag(111) surface; the breakthrough is achieved using the MBE procedure to provide precise control. The atomic and electronic structures of P chains are determined by in situ scanning tunneling microscopy/spectroscopy (STM/STS) operated at 77 K, low energy electron diffraction (LEED), and high-resolution angular photoemission spectroscopy (HR-ARPES). Large areas of high-quality P chains are successfully synthesized, with a measured band gap of 1.8 eV. X-ray photoelectron spectroscopy (XPS) analysis reveals a weak interaction between the P chains and the Ag(111) substrate, which precludes the existence of any potential silver–phosphorus alloy. The STM topography of the P chains and the band gap obtained in the spectroscopy are confirmed by density functional theory (DFT) calculations. The atomically precise synthesis of P chains represents a breakthrough toward the rapid integration of P chains in real devices by overcoming the limitations of the top–down approach to P chain synthesis from black phosphorus. The growth is performed in an ultra-high vacuum (UHV) apparatus equipped with the standard tools for growth and characterization (see “Methods”).

## Results

**Fig. 1:**
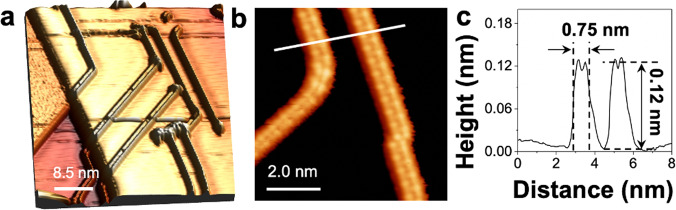
Formation of P chains from the early stage of growth. **a** 3D view of the STM topographic image of P chains on the Ag(111) surface, showing atomically flat terraces and P chains (46 × 46 nm^2^, *U* = −800 mV, *I* = 1.50 nA). **b** High-resolution STM image of P chains on the Ag(111) (7 × 7 nm^2^, *U* = −80 mV, *I* = 1.50 nA). **c** Line profile measured along white line in **b** shows that the P chains have a typical width of 0.75 ± 0.01 nm and a height of 0.12 nm.

**Fig. 2:**
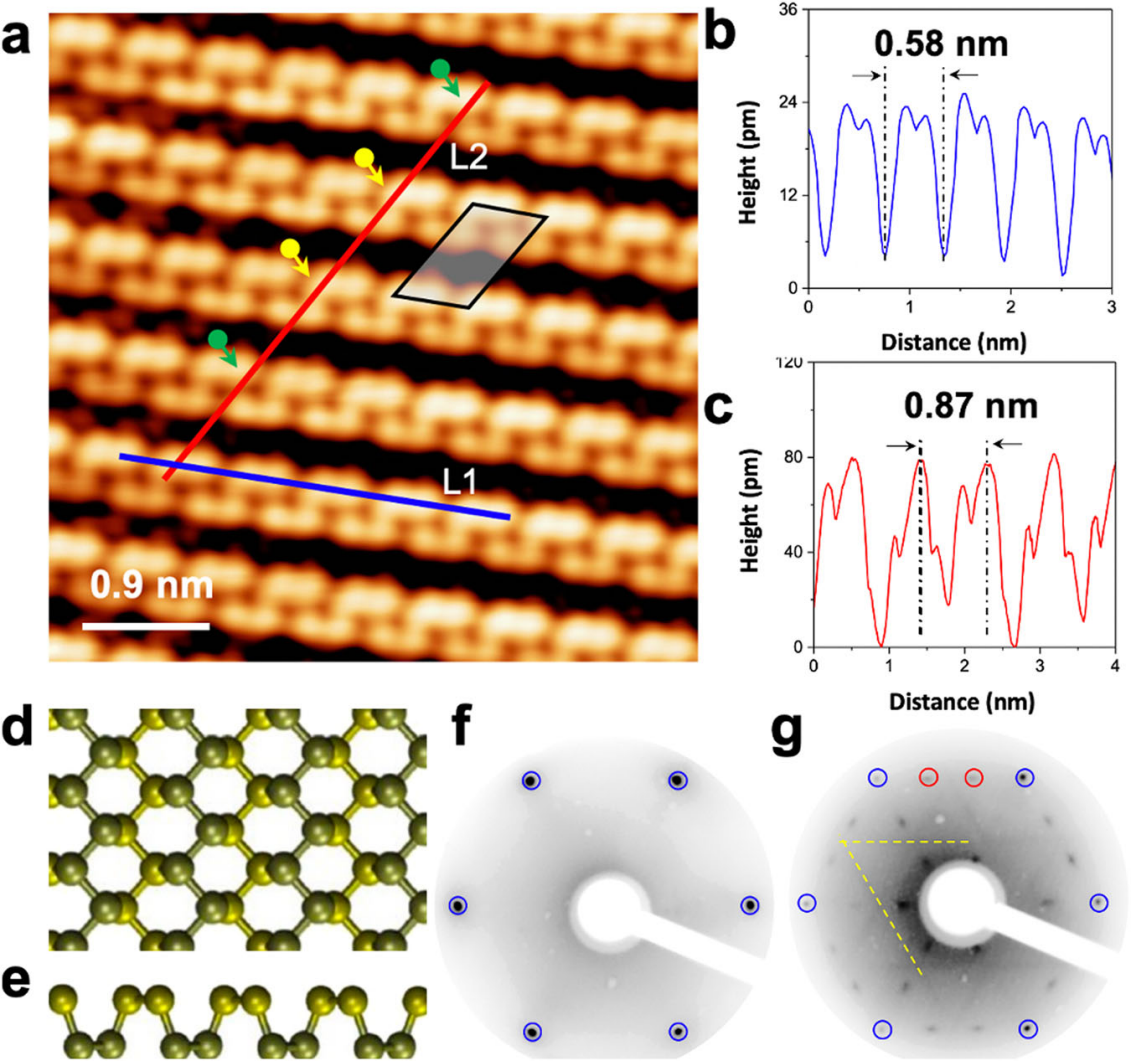
Atomic structures of P chains on Ag(111). **a** Atomically resolved STM image, showing parallel P chains (5 × 5 nm^2^, *U* = −0.1 V, *I* = 1.00 nA). The unit cell of P chain structure is indicated by the black rhombus. **b** Line scan measured along the line L1 in **a**. The periodicity of the brightest protrusions is around 0.58 ± 0.01 nm, corresponding to twice the lattice constant of the Ag(111) surface (2 × 0.289 nm = 0.578 nm). **c** Line scan measured along the line L2 in **b**. The line profile L2 measures the corrugation across the P chains along the other main direction of Ag(111) in **a** and shows a periodicity of 0.87 ± 0.01 nm, which is equal to three times the lattice constant of Ag(111) surface (3 × 0.289 nm = 0.867 nm). **d**, **e** Structural model of black phosphorene layer viewed from the top and side, respectively. **f**, **g** Low energy electron diffraction patterns recorded at 56 eV corresponding to the bare Ag(111) and after deposition of ~0.8 ML of P on Ag(111), respectively. The (1 × 1) spots of Ag substrate are highlighted by blue circles, the ×3 are highlighted by red circles, and the ×2 are highlighted by dashed yellow lines.

Figure 1a presents a large topographic STM image corresponding to low-coverage deposition of P atoms (~0.1 ML) on the Ag(111) surface. This image shows different atomically flat Ag(111) terraces, covered by isolated P chains. Their number depends on the P coverage; at low coverage, only a few chains are observed, while at higher coverage, the P chains assemble into 2D arrays (Fig. 2a). A number of examples in the literature support this coverage-dependent behavior: silicene on Ag(110)^48^, silicene on Au(110)^49,50^, and Si on SiC(001)^51^. We observe that, at the earliest stage of growth, P atoms are not disordered but the P chains are formed rapidly. Figure 1b (zoom of Fig. 1a) presents an atomically resolved STM image of individual P chains. The P chains have the same internal atomic structure, consisting of two rows of bright protrusions, with a width of 0.75 ± 0.01 nm and a height of 0.120 ± 0.001 nm (line profile in Fig. 1c). The P chains lie on top of the Ag(111) surface and are aligned along the main crystallographic directions; a few kinks and bends are visible.

After deposition of ~0.8 ML of phosphorus on Ag(111), a high density of self-assembled P chains with different domains is visible in the large-scale STM image (Supplementary Fig. 1a). The lengths of the P chains were analyzed from the STM images taken at random positions on the surface. Supplementary Fig. 1b shows a histogram of the distribution of the chain lengths. Despite the finite image size that truncates the longer P chains^52^, their average length is around 25 nm. The fine structural details in the atomic-resolved STM image in Fig. 2a reveals an armchair-like structure of the P chains. The distance between the first nearest-neighbor protrusions is 0.21 ± 0.01 nm, close to that of BP, while the corrugation between neighboring P atoms is very small (<10 pm), indicating that all the atoms have the same height with respect to the surface plane. The line profile of Fig. 1c shows that the P chains have the height of a single layer.

In that respect, the interaction with the Ag(111) surface causes the natural buckled chain structure to be flattened in the armchair direction. The dimensions of the unit cell are 0.58 nm × 0.87 nm from the line profiles L1 and L2 (Fig. 2b, c, respectively). The unit cell dimensions and line profiles indicate that the P chains self-assemble to form a (2 × 3) superstructure relative to the Ag(111) surface. Note that the 2 × 3 periodicity of P chains is not perfect because some P chains are shifted laterally relative to the neighboring nanowires by one Ag lattice parameter as indicated in Fig. 2a by the green and yellow arrows, respectively.

A comparison of the atomic-resolved STM image resembles remarkably the ball and stick model (Fig. 2d) of a black phosphorene layer viewed from the side (Fig. 2e), suggesting that the obtained structure corresponds to flat P chains with an armchair structure. If we consider the projection of the side view (Fig. 2e) of the black phosphorene, it has the armchair structure (two up atoms and two down atoms) due to the puckered arrangement of the P atoms. In this “phosphorene-like” form, these armchair chains of phosphorene atoms lie in the plane parallel to the surface. Hence, this observed form of phosphorene is flat.

The surface was also characterized using LEED. The LEED patterns were recorded at 56 eV, on the bare Ag(111) surface (Fig. 2f) and after deposition of ~0.8 ML of P (Fig. 2g). In both images, the diffraction spots corresponding to the Ag(111) surface are indicated by blue circles. Following the deposition of P atoms, different spots appear corresponding to a ×3 structure (red circles) with respect to the silver substrate in good agreement with the STM observations. The elongated spots of the ×3 structure indicate that the order is not perfect along this direction. This is due to the shift between two neighboring P chains that we see in the STM images (Fig. 2a). The thin elongated streaks with a ×2 periodicity appear weak in the diffraction pattern (outlined by the  yellow dashed line). This weakness is due to an inter P chain shift of one atomic parameter of silver. This behavior was also observed in the case of silicene NRs on Ag(110)^50^.

**Fig. 3:**
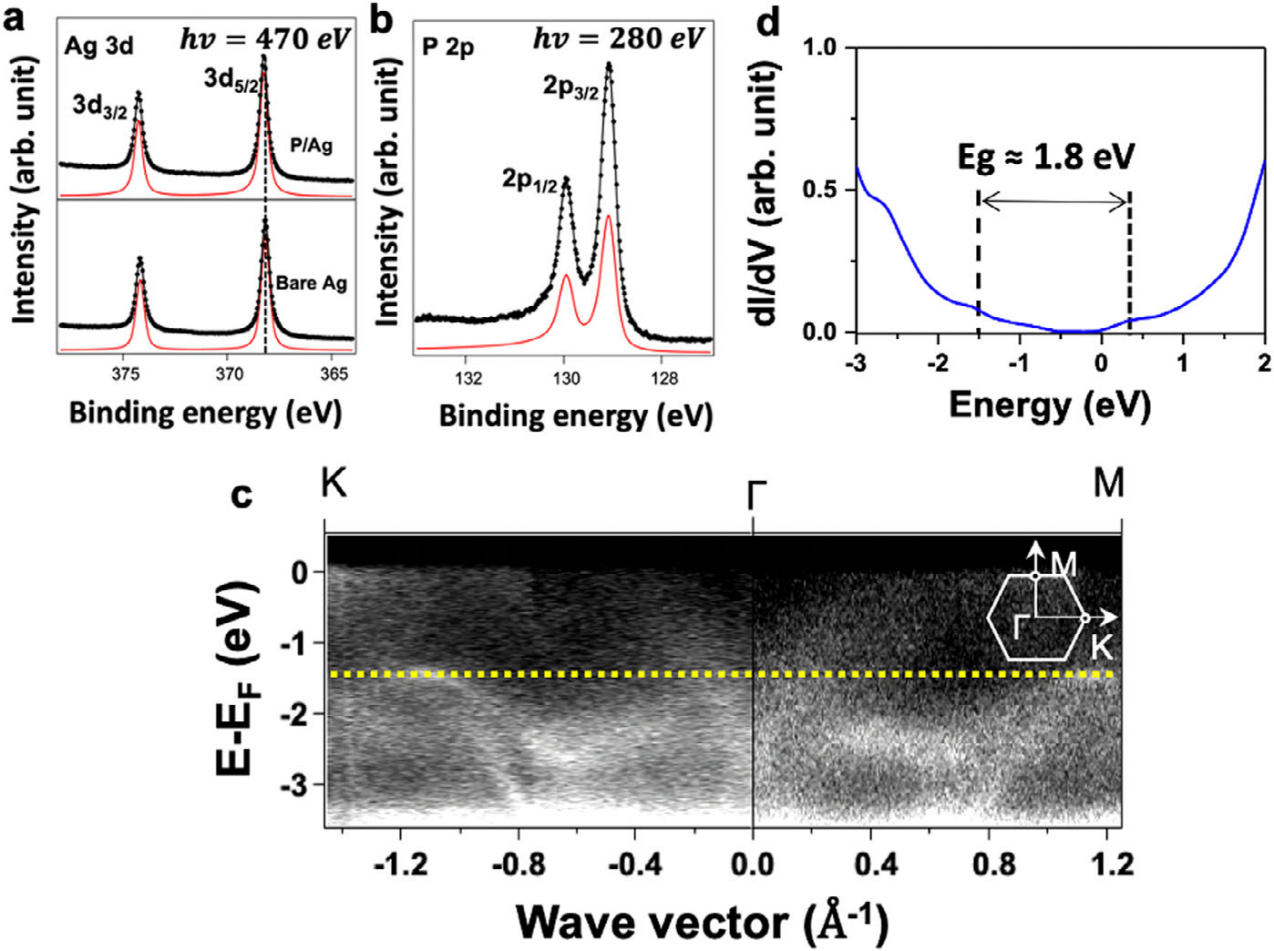
Experimental XPS, HR-ARPES, and simulated spectra of P chains on Ag(111). **a** XPS spectra of Ag 3*d* core levels before (lower panel) and after growth of P chains (upper panel). **b** P 2*p* core-level spectrum, presenting only one doublet (red curve). (Black dots present the data; black curve presents the best fit.) **c** The HR-ARPES were recorded at photon energy of 60 eV along *K*–Γ–*M* directions after deposition of P chains on Ag(111) surface. **d** STS d*I*/d*V* spectra acquired on the P chains, demonstrating the semiconducting character of P chains. The curve is an average of 40 individual spectra.

The chemical environment at the interface between the as-grown P chains and the substrate was characterized by XPS measurements. Figure 3a shows the Ag 3*d* core-level spectra corresponding to the bare substrate and after deposition of ~0.8 ML of P atoms, respectively. For both spectra, the characteristic Ag 3*d*_3/2_ and 3*d*_5/2_ peaks are located at the same binding energies of 374.2 and 368.2 eV, respectively (see “Methods”). The binding energy values and their shapes remain unchanged. We also observe no detectable peak splitting nor any significant energy shifts after the phosphorus deposition, pointing to a weak interaction between the P chains and the Ag surface. The P 2*p* core-level spectrum presented in Fig. 3b reveals only one spin–orbit doublet indicating that P atoms have only one chemical environment. The 2*p*_1/2_ and 2*p*_3/2_ peaks are located at 129.89 and 129.03 eV, respectively. In addition, no peak is detected at higher binding energy, which would correspond to oxidized phosphorus^53^. This indicates that the as-grown P chains on the Ag(111) are composed of pure phosphorus with a high structural quality. The STM and XPS results concord that the P chains are flat on the surface with an armchair-like structure in which the P atoms have the same chemical environment.

The electronic structure of the P chains is also explored through a combination of HR-ARPES and STS measurements. Figure 3c shows the band structure of the P chains obtained by HR-ARPES. Measurements were recorded parallel to and perpendicular to the armchair direction. In reciprocal space, this corresponds to the Γ–*K* and Γ–*M* directions, respectively. A dispersive band associated with the phosphorene layer is located at 1.50 ± 0.20 eV below the Fermi level, indicating unambiguously the existence of an energy gap of at least 1.5 eV. Despite the 1D character of the P chains, we measure here the electronic band structure associated with the 2D network of P chains adsorbed on the Ag(111) surface. This explains the observation of electronic bands in the direction perpendicular to the chains (Γ–*K*). This type of peculiar band was observed on an exfoliated single layer of black phosphorus and was explained by the contribution from a bonding and anti-bonding pair of the P 2*p*_*z*_ orbitals^54^. These bands are observed here on an epitaxial phosphorene  ML and there is almost no influence of the underlying Ag substrate, which reflects a very weak interaction at the interface.

Figure 3d shows a typical STS d*I*/d*V* spectrum recorded on the P chains presenting a clear semiconducting character. At negative bias, the local density of states (LDOS) is flat and featureless between the Fermi level and *U*_gap_ = −1.5 V. The *sp* state of the underlying silver contributes a slight rise in the DOS between −0.7 and −1.5 V but is strongly attenuated by the P chains. This behavior is seen in the case of NaCl/Ag^55,56^, and as we describe below, the band folding within the 2 × 3 unit cell induces a background in the spectra. The peak located at −1.5 V is attributed to the valence band maximum, consistent with the HR-ARPES observation (horizontal yellow dashed line in Fig. 3c). At positive bias, the conduction band minimum is located at *U*_gap_ = +0.3 V above the Fermi level. Combining the STS and HR-ARPES gives a band gap close to 1.80 ± 0.20 eV for the P chains.

**Fig. 4:**
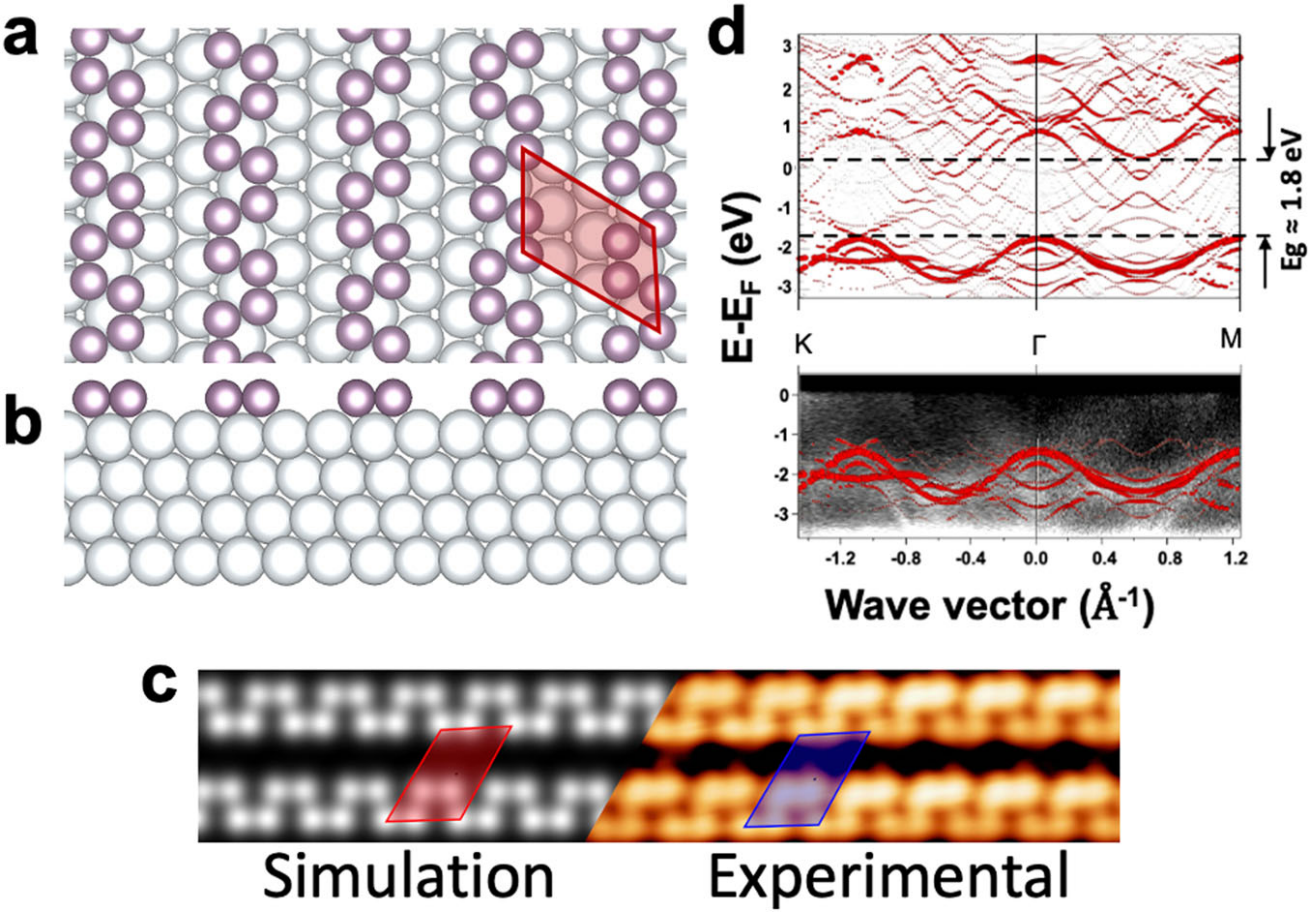
Model of PNR structure from DFT calculations. **a** Top view showing the armchair PNR structure. P atoms are in purple, Ag atoms in gray. The 2 × 3 unit cell is indicated by the red parallelepiped. **b** Side view showing the atoms of the P chains lying flat on the Ag substrate. **c** Simulated (left) and experimental (right) STM images. **d** (upper panel) The calculated DFT band structure of the whole 4P/(2 × 3)-Ag(111) surface along the *K*–Γ–*M* path of the (1 × 1)-Ag(111) surface using 160 k-points. **d** (lower panel) The superposition of the experimental and the calculated ARPES shows an excellent agreement.

To confirm the experimental observations, we performed DFT calculations (see “Methods”). After full relaxation of the system, the resulting structure is composed of an ordered array of phosphorene  chains (Fig. 4a, b), closely resembling the STM observations (Fig. 2a). We find a binding energy of 0.71 eV per P atom, reflecting an interaction that can be described as either weak chemisorption or a strong physisorption. The calculated corrugation of P chains is negligible (0.004 nm) and in excellent agreement with the STM results. The calculated nearest-neighbor distance in the P chains was found to be 0.22 nm in close agreement with observation (0.21 nm). We simulated the STM image from the partial charge density of the relaxed systems using the Tersoff–Hamann approach (see “Methods”). Figure 4c shows side by side the experimentally determined image (right) and the computed image (left). The calculated STM image from the proposed model is in excellent agreement with the experimental STM image.

To complement this, we also calculated the electronic band structure of the system (Fig. 4d). We present here the contribution of P chains of the full P/Ag(111) system but the contribution of P and Ag cannot be decoupled experimentally. The contribution of the P atoms is superimposed on the experimental HR-ARPES. The results fit the experimentally observed dispersion band very well (Fig. 3c). Obviously using *k*-points from the first Brillouin zone of the (1 × 1)-Ag(111) unit cell induces the appearance of several folded bands. However, one can observe the standard parabolic dispersion of silver around the Γ point. In addition, the P contribution at 1.60 ± 0.20 eV below the Fermi level is clearly visible and is close to the experimental value (1.5 eV). From the calculations, the band gap equals 1.80 eV in good agreement with STS measurements.

Further calculations and analysis are presented in the Supplementary Information, in particular the calculated band structure of an isolated P chain without the Ag substrate exhibiting a metallic character (Supplementary Fig. 2). In comparison, Supplementary Fig. 3a, b shows the band structure and the DOS of a hydrogenated P chain, respectively. The hydrogenation leads to the opening of a band gap in the electronic structure. These calculations support the fact that the Ag substrate passivates the P chains. Interestingly, the band gap is indirect on Ag, compared to H-passivated free-standing phosphorene, which might be attributed to variations in the local charge transfer. Moreover, we have probed the interaction between the P chains in the bi-dimensional array by calculating the DOS of the isolated P chain (in a large Ag unit cell) compared to the chain in the array (Supplementary Fig. 4). The DOS from these two calculations are very similar, which indicates that there is no direct interaction between the chains, rather an indirect one through the Ag substrate.

The band structure of the bare 2 × 3-Ag(111) unit cell has also been calculated (Supplementary Fig. 5), showing in conjunction with Supplementary Fig. 2 that the full band structure does not result in the simple superposition of the band structures of the isolated systems. Besides, it is rather difficult to interpret the features above the yellow line in Fig. 3d because of the band folding of the silver surface, which gives rise to an electronic background covering all this specific area of the Brillouin zone. In addition, spin-polarized calculations have been performed showing no magnetism in the P chain (Supplementary Fig. 8). Finally, ab initio molecular dynamics simulations have been performed at 500 K to probe the stability of the P chains on the Ag surface. The results shown in Supplementary Figs. 9 and 10 reveal a strong stability of the system in agreement with the experiments.

In summary, we present a controlled synthesis of flat phosphorene-like P chains on Ag(111) using the MBE process. The atomic and electronic structures of these epitaxial P chains were experimentally determined and confirmed by DFT calculations. The P chains self-assemble on Ag(111) surface and form an armchair-like structure giving a (2 × 3) superstructure. They are semiconducting with a band gap of 1.8 eV. The discovery of P chains on Ag(111) using the bottom–up approach opens the way for rapid technological advances based on phosphorene. Our contribution concerns a first step along this road, namely, the fabrication of well-defined 1D P chains by MBE methods, which in itself constitutes a breakthrough. Our STM study shows that the 1D P chains have a uniform armchair structure and are semiconducting. Nevertheless, realistically several challenges remain; the first is the non-negligible interaction of the 1D P chains with the Ag substrate. This can be overcome by hydrogenation of the P chains^39,57^ or growth on a thin NaCl layer^56^. In these two examples, one can anticipate the P chains to show a direct electronic band gap. This would enable the excitonic structure of the P chains to be investigated by STM luminescence as has been demonstrated recently on MoSe_2_ flakes^58^.

## Methods

### Experiments

The experiments were carried out with a commercial UHV system, consisting of three inter-connected chambers, where the base pressure is better than 3.0 × 10^−10^ mbar. The single-crystal Ag(111) substrate (99.999%) was cleaned by several cycles of Ar^+^ sputtering and post-annealing. The beam energy is 2 keV at 5 × 10^−6^ mbar and the subsequent annealing temperature of 500 °C is maintained for 40 min to remove any contamination. A Knudsen cell loaded with commercial bulk black phosphorus was used as a phosphorus source to grow the phosphorene chains. A series of experiments was performed with various phosphorus coverages corresponding to deposition times ranging from 1 to 8 min, in which high-purity phosphorus was deposited onto Ag(111) while the substrate was kept at RT. During growth, the Knudsen cell was heated to 450 °C giving a stable deposition rate of 0.1 ML/min, calibrated by a quartz microbalance. The surface structure and chemical composition were monitored by LEED and AES measurements performed at RT. The morphology of sample was characterized by in situ low-temperature STM, operated at 77 K. All STM measurements were recorded at a constant current mode. The tungsten (W) tips were made by electrochemical etching. STS experiments were measured at 77 K using a standard lock-in technique, where a modulation voltage of 10 mV at a frequency of 5127.7 Hz was applied.

The photoemission experiments were performed using the same substrate on the TEMPO beamline of the Synchrotron SOLEIL-France. The photon energies of the XPS experiments were recorded at 470 ± 0.20 eV for Ag 3*d* and 280 ± 0.20 eV for P 2*p*. All spectra are fitted with a Doniach–Sunjic line shape^59^. The best fit of Ag 3*d* was obtained with a 140 meV Gaussian profile and a 280 meV Lorentzian profile, while the spin–orbit splitting is 6 eV. The best fit of P 2*p* was obtained with a 200 meV Gaussian profile and a 80 meV Lorentzian profile, while the spin–orbit splitting is 0.865 eV.

### Calculations

The present calculations were performed using DFT. In the calculations, we considered a 2 × 3-Ag(111) slab of 5 layers on top of which 4 phosphorous atoms were positioned according to the standard geometry of the side view of a black phosphorus layer. To test the size effect, a calculation using nine layers was performed. Correlation effects were treated using the Perdew–Burke–Ernzerhof exchange–correlation functional^60^ as implemented in VASP^61–63^ version 5.4.4. The interaction between the valence electrons and ionic cores was described by the projector augmented wave method^64,65^. The plane wave energy cut-off was set to 400 eV. We then optimized the geometry until the residual forces fell <0.01 eV/Å. The calculated STM images were produced using the Tersoff–Hamann method^66^ and the p4vasp software package with an STM tip placed 2.05 Å above the position of a phosphorus atom.

## Supplementary information

Supplementary Information

## Data Availability

All data needed to evaluate the conclusions of this study are available in the main text or Supplementary Materials. The data that support the findings of this study are available from the corresponding authors upon reasonable request.
