# Supplementary material for: Flat epitaxial quasi-1D phosphorene chains
Source: Nat Commun. 2021 Aug 27;12:5160. doi: 10.1038/s41467-021-25262-7 (PMC8397792; doi:10.1038/s41467-021-25262-7)
Supplement: Supplementary file 1 — Supplementary Information [file 41467_2021_25262_MOESM1_ESM.docx]

Supplementary Information

Flat epitaxial quasi-1D Phosphorene chains

Wei Zhang^1^, Hanna Enriquez^1^, Yongfeng Tong^2^, Andrew J. Mayne^1^, Azzedine Bendounan^2^, Alex Smogunov^3^, Yannick J. Dappe^3^, Abdelkader Kara^4^, Gérald Dujardin^1^, and Hamid Oughaddou^1,5^**^*^**

^1^Université Paris-Saclay, CNRS, Institut des Sciences Moléculaires d’Orsay, 91405 Orsay, France

^2^TEMPO Beamline, Synchrotron SOLEIL, L’Orme des Merisiers Saint-Aubin, B.P.48, F-91192 Gif-sur-Yvette Cedex, France

^3^Université Paris-Saclay, CNRS, CEA, Service de Physique de l'Etat Condensé, 91191 Gif-sur-Yvette, France

^4^Department of Physics, University of Central Florida, Orlando, FL 32816, USA

^5^Département de physique, CY Cergy Paris Université, F-95031 Cergy-Pontoise Cedex, France

**^*^**e-mail: [Hamid.oughaddou@universite-paris-saclay.fr](mailto:Hamid.oughaddou@universite-paris-saclay.fr)

**Supplementary Text**

After deposition of ~ 0.8 monolayer (ML) of phosphorus on Ag(111), a much higher density of phosphorene-like P chains is visible in the large-scale STM image presented in Supplementary Figure 1 (a). The P chains grow on the flat Ag(111) terraces with a high degree of alignment between neighboring P chains compared to that seen at low-coverage (Supplementary Figure 1 (a)). The growth directions of the three domains are aligned with the Ag(111) substrate as the atomically-resolved STM image of the bare Ag(111) in the inset of Supplementary Figure 1 (a) highlights.

To determine the lengths of the P chains, we analyzed the STM images taken at random positions on the surface and measured the lengths of every chain. Supplementary Figure 1 (b) shows the histogram of the distribution of their lengths. The typical length of the P chains is around 25 nm.


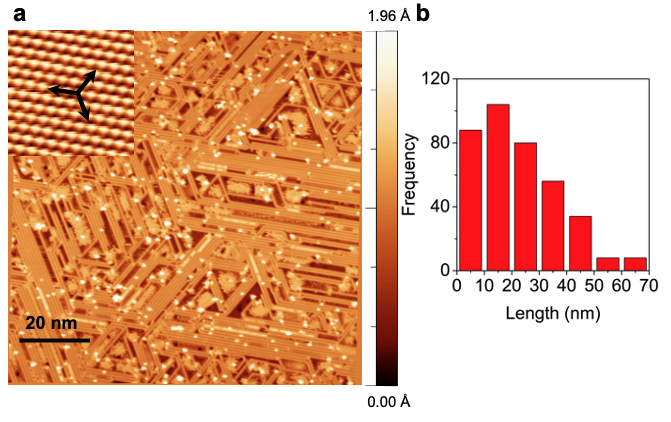


Supplementary Figure 1. **Atomic structures of P chains on Ag(111).** (a) Large-area (100 x 100 nm) STM image of ~0.8 ML of P on the Ag(111) surface (U = −1.0 V, I = 1.0 nA). The inset shows an atomically resolved STM image of the clean Ag(111) surface. The black arrows indicate the principle crystal directions of the Ag surface. (b) Histogram showing the distribution of lengths of the P chains measured in the image

We have calculated the band structure of the free-standing P chains without the Ag substrate. The dispersion of the electronic bands is consistent with a metallic structure (Supplementary Figure 2).


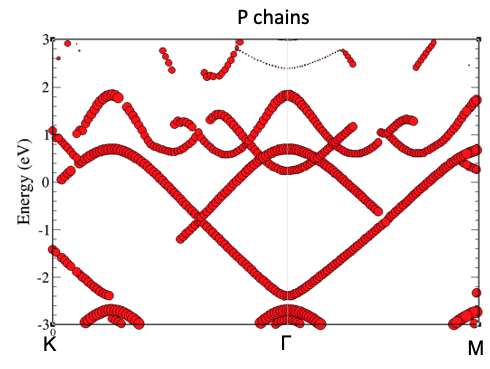


Supplementary Figure 2. DFT-calculated band structure of the free-standing P chains (without Ag substrate), exhibiting a metallic character of the chains.

In addition, we have calculated the band structure for free-standing chains that are passivated with H atoms (Supplementary Figure 3a). Again, without the substrate, in this case, the H-P chains show a semiconductor behavior. We also observe in the band structure plot (Supplementary Figure 3 a), that the band gap is direct as predicted by theory. The corresponding calculated DOS of the passivated P chains is shown in Supplementary Figure 3 b.


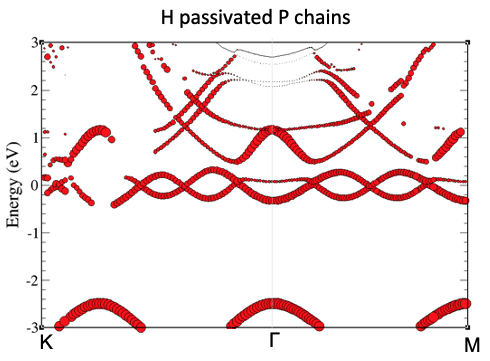

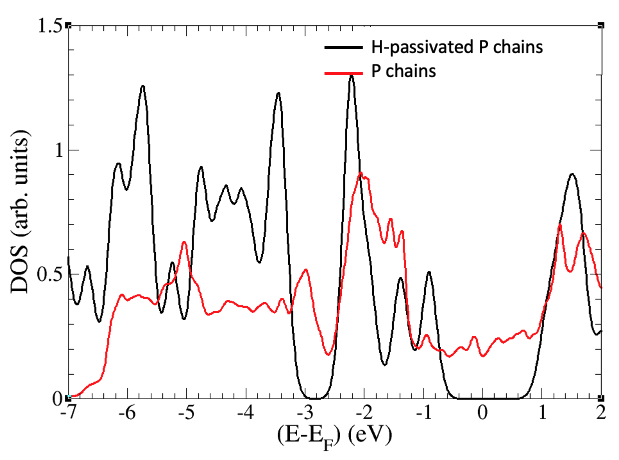


(a) (b)

Supplementary Figure 3**.** (a) DFT-calculated band structure of free-standing P chains passivated with hydrogen atoms, exhibiting a semiconductor behavior. (b) Corresponding DFT-calculated DOS of the H-passivated P chains, compared to the same P chains without passivation.

These calculations all support the fact that Ag substrate passivates the P chains (see Figure 3c and Figure 4d in the main article), leading to the opening of a band-gap in the electronic structure, as is found in H-passivated free-standing phosphorene. However, the band gap is indirect compared to H-passivated free-standing phosphorene, which might be attributed to variations in the local charge transfer.

We have performed extra calculations to isolate properly the 1D nano structure by using a bigger Ag unit cell while keeping only one P chain within the unit cell. Thus, Supplementary Figure 4 presents the calculated DOS for isolated P chains (large cell) or a self-assembled array of 1D P chains, which happen to be almost identical. The very similar adsorption energies (Eads = 0.75 eV for isolated chains and 0.71 eV for the array) indicates that there is no direct interaction between neighbouring 1D P chains, only via the Ag substrate.


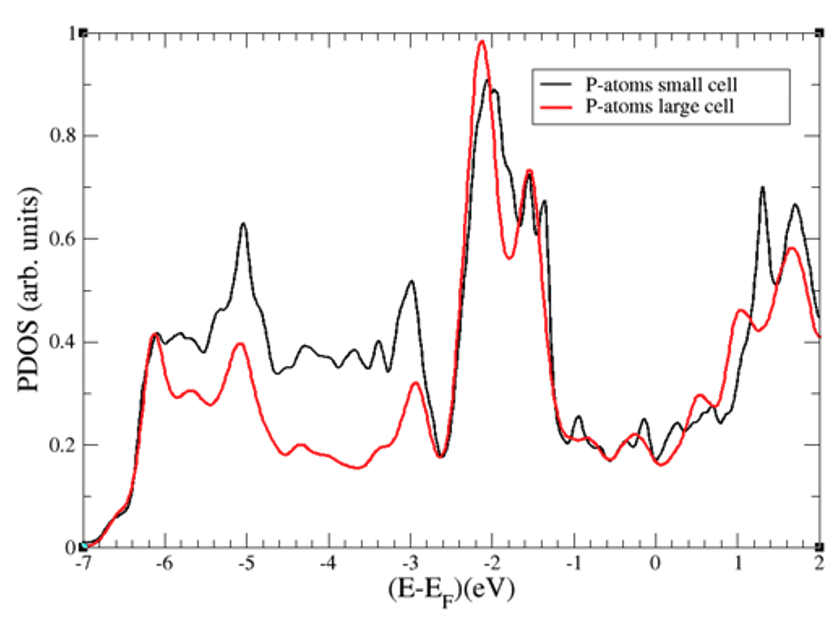


Supplementary Figure 4. Comparison of the projected DOS of the P chains in a 2x3 Ag(111) unit cell (small cell in black) and in a 2x9 Ag(111) unit cell (large cell in red), calculated in DFT using the PBE functional.

We calculated the band structure of the bare Ag substrate (without the P chains, Supplementary Figure 5). As a result, it can be clearly seen that the band structure of the full system is not the simple superposition of the band structures of each isolated subsystem. Indeed, due to the passivation of the P chains by the silver atoms, the P 1D chains become semiconducting upon adsorption on the Ag(111) surface (See Figure 4d) whereas the isolated 1D chains are metallic. On another hand, the Ag(111) surface is rather complicated to characterize due to the important band folding within the 2x3 unit cell


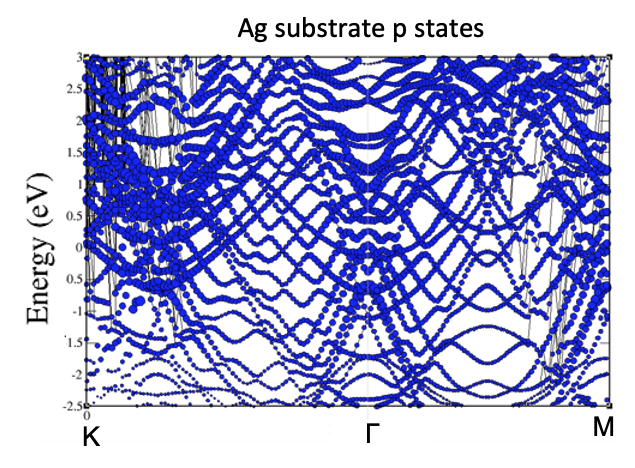


Supplementary Figure 5. DFT-calculated band structure of the clean 2x3 Ag(111) unit cell (without P atoms) along the same path in the Brillouin zone as in Figure 4 (d) in the manuscript.

We present in Supplementary Figure 6 the second derivative plot of the ARPES band mapping obtained on P chains on Ag(111) that we reported in Figure 3 of the manuscript. The bands associated with the P chains are clearly seen in Supplementary Figure 6 and they confirm the existence of a band gap of at least 1.5 eV. As indicated in the Figure, the other dispersive bands correspond to the Ag *sp* states.

**
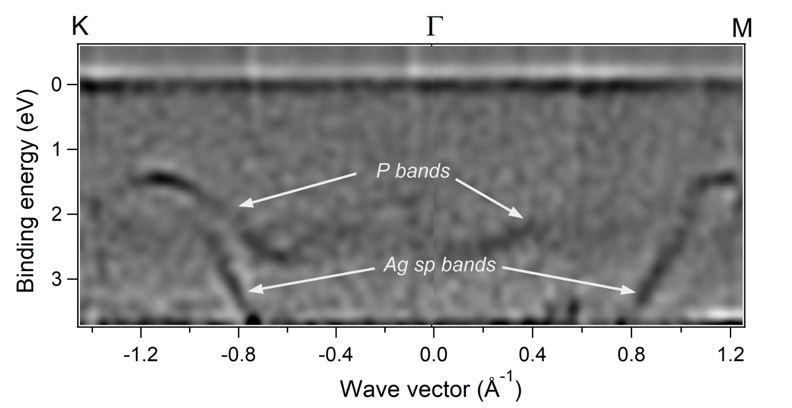
**

Supplementary Figure 6. Second derivative plot of the ARPES data obtained on P chains grown on Ag(111).

We have also calculated the PDOS using PBE and HSE functionals for P chains in the 2x3 Ag(111) unit cell. The calculations using the hybrid functional HSE06 show only small differences compared to the PBE calculations; the values of the gap are very close, despite a small change in the overall doping of the ribbon. We added in the Supplementary information the calculated DOS for gas-phase and adsorbed P chains using both PBE and HSE06 (Supplementary Figure 7)


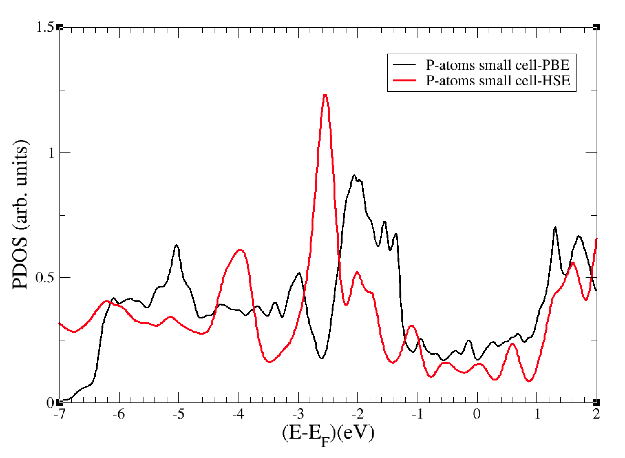


Supplementary Figure 7. Comparison of the P chains PDOS calculated using PBE and HSE functionals for chains in the 2x3 Ag(111) unit cell

We have also performed spin-polarized calculations on the P chain on Silver. The Supplementary Figure 8 show clearly no spin-polarization in the system, meaning that these P chains are non-magnetic.


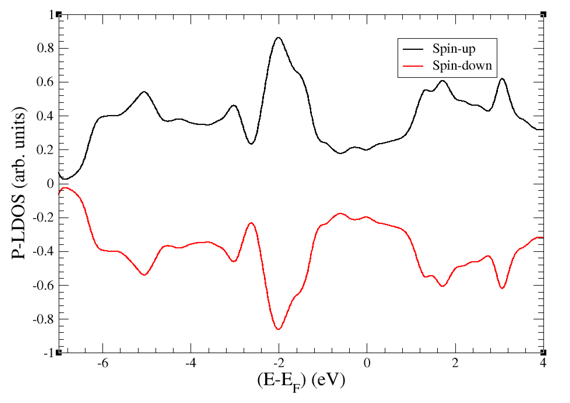


Supplementary Figure 8. Spin-polarized PDOS of the P chains in the 2x3 Ag(111) unit cell.

We performed *ab initio* molecular dynamics (AIMD) simulations as implemented in VASP. The simulations were performed at 500K in the NVT ensemble and using the Nose-Hoover thermostat^1^ to control the temperature of the system. The system consisted of 4 P atoms and 24 Ag atoms (6 Ag per (111) layer). The bottom 2 layers were kept fixed. The time step was set to 1 fs. We first performed a 5ps run to thermalize the system followed by a 10ps production run. Because our system is very small (only 28 atoms, where only 16 are allowed to move), the fluctuations in the temperature are quite large. The Supplementary Figure ~~9~~ shows the temperature fluctuations during the 10ps simulation run.


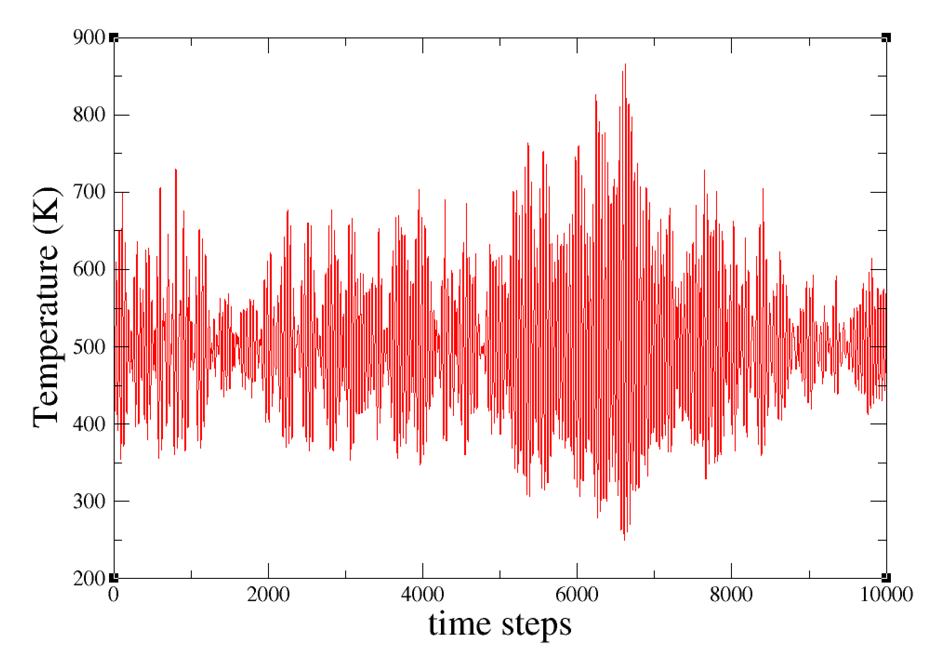


Supplementary Figure 9. Temperature of the system during the NVT simulation

We have monitored the atomic positions of all the phosphorus atoms during the 10ps and found that these atoms were oscillating around their equilibrium position. In Supplementary Figure 10, we show snapshots of the system between 1ps and 10 ps, at intervals of 1ps. We note that the system is very stable at 500K during the whole 10ps simulation time.

| 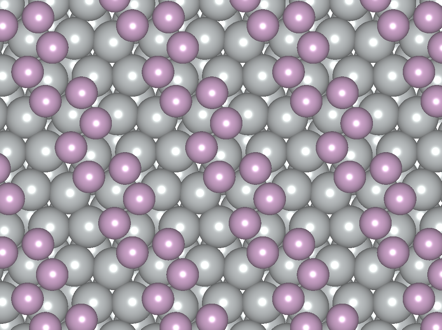  1ps | 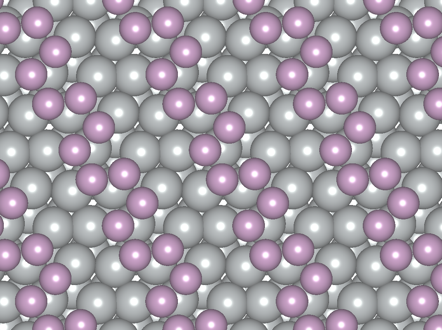  2ps |
| --- | --- |
| 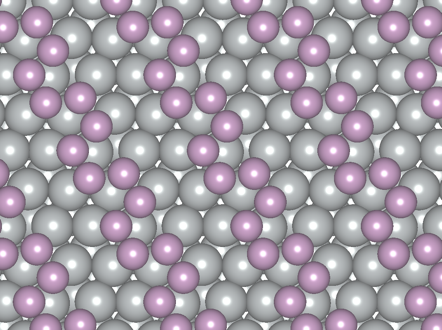  3ps | 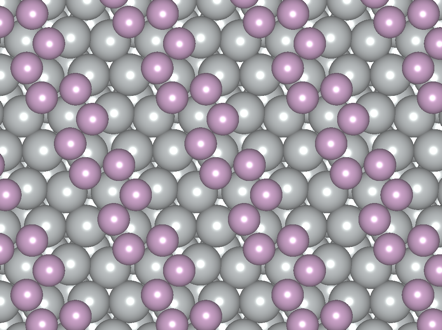  4ps |
| 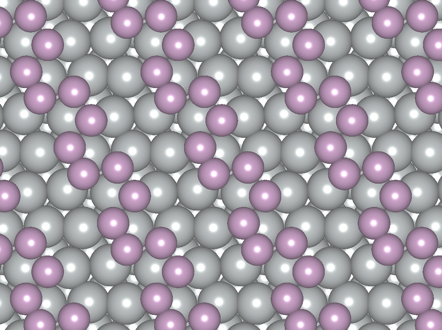  5ps | 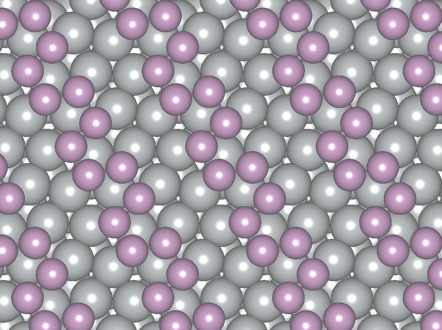  6ps |
| 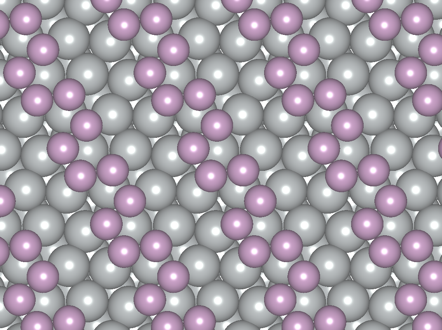  7ps | 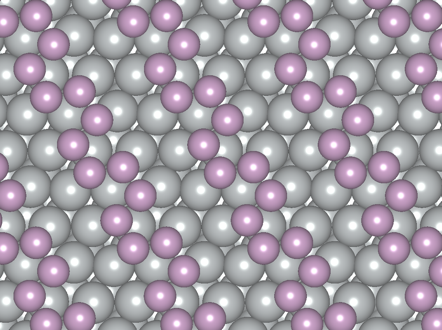  8ps |
| 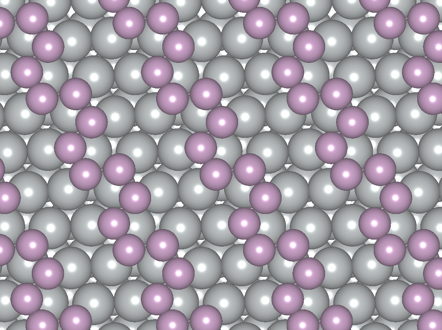  9ps | 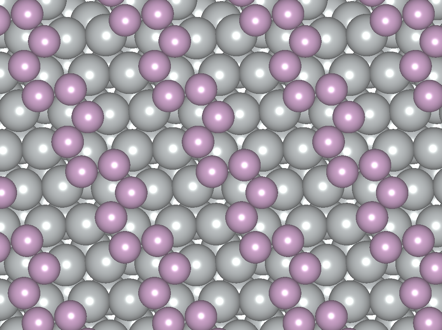  10ps |

Supplementary Figure 10. A series of snapshots of the system at 500K at 1ps intervals.

**REFERENCES**

^1^Nosé, S. A unified formulation of the constant temperature molecular dynamics methods. J. Chem. Phys. 81, 511 (1984).
